# Social isolation and multiple chronic diseases after age 50: A European macro-regional analysis

**DOI:** 10.1371/journal.pone.0205062

**Published:** 2018-10-24

**Authors:** David Cantarero-Prieto, Marta Pascual-Sáez, Carla Blázquez-Fernández

**Affiliations:** Group of Health Economics and Health Service Management, Department of Economics, The University of Cantabria–IDIVAL, Santander, CP, Spain; Indiana University Purdue University at Indianapolis, UNITED STATES

## Abstract

**Background:**

Different studies have found that socioeconomic determinants influence the prevalence of chronic diseases in older people. However, there has been relatively little research on the incidence of how social isolation may affect them. We suggest that social isolation is a serious concern for people living with chronic illnesses.

**Method:**

In this paper, we examine whether there is an increase in the propensity of being diagnosed with chronic illnesses because of a decrease in social relations for elderly Europeans. We have used a panel data for Waves 1–6 (2004–2015) of Survey on Health, Ageing and Retirement in Europe (SHARE) and logistic regressions. Besides, we have studied three geographic macro-areas (Nordic, Continental and Southern). Being diagnosed with three or more chronic diseases is considered as a dependent variable, and as social control variables we have used three isolation proxies (living alone, providing help to family, friends or neighbours and participation-club activities). Other socio-demographic variables are included (gender, age, educational level, job situation, area of location and quality of life).

**Results:**

Our results for the full sample indicate that people who participate in social activities have fewer probability of suffering from chronic diseases (OR = 0.70, 95% CI 0.54, 0.92). For people who live alone the reverse effect is observed (OR = 1.20, 95% CI 1.04, 1.39). Differences are shown by macro-areas, e.g. providing help (OR = 0.58, 95% CI 0.34, 0.97) isolation proxy is significant for the Nordic macro-area. Club-participation activities and living alone are significant for Continental and Southern macro-areas, respectively (OR = 0.65, 95% CI 0.55, 0.82; OR = 1.46, 95% CI 1.21, 1.77).

**Conclusions:**

Social isolation increases the risk of being diagnosed with chronic illnesses. That is, people with greater social participation have lower risk of suffering from multiple chronic diseases. This risk linked to isolation, together with the traditional one associated with lifestyles, should be considered in the development of new public policies.

## Introduction

In accordance with the World Health Organization [1] *“health is a state of complete physical*, *mental and social well-being and not merely the absence of disease”*. Furthermore, in a profuse understanding of health it should be highlighted that it is related with physical, social and economic circumstances [2–4]. Besides, as advanced by Lago et al. [5], the published literature on socioeconomic status, health and Non-Communicable Diseases (NCDs) is characterized by many papers postulating the complexity of these relationships. Correspondingly, it advocates that further research is necessary regarding those different factors related with health status, particularly, in NCDs [6].

Chronic diseases are characterized by long duration, and usually, slow progression. NCDs cause more deaths than the combination of other causes. Indeed, it is estimated that NCD deaths will have increased from 38 million in 2012 to 52 million by 2030 [7]. Among the leading risk factors for NCDs, in general, are high blood pressure, tobacco, high blood glucose, physical inactivity, obesity, high cholesterol, and alcohol consumption. However, in addition to these behaviour-related factors, social and economic variables such as poverty, inequality or social displacement are latent [8]. Among the aforesaid factors, in this manuscript, we focus on social isolation (referring therefore to contacts with individuals within the respondent’s network, and not taking as such an individual’s subjective feeling of loneliness). In this regard, it should be mentioned that there is a vast amount of research on social isolation and health. Social isolation has been associated with mortality and morbidity, see for example UK Biobank Studies in Lancet Public Health [9] and in Heart [10], and a recent review by Holt-Lunstad [11] that discusses the current literature with a broader perspective. That is, social isolation has social and health implications [12–13]. In this regard, although individual socioeconomic status has been linked with chronic diseases [14], there has been relatively little research into the question of how social isolation may affect multicomorbidity [15–17]. More research is thus needed to better understand social isolation and multiple chronic diseases.

Our objective is to study whether there is an increase in the propensity of being diagnosed with chronic illnesses because of social displacement after age 50. Social isolation becomes an important risk at older ages because several events occur at the same time: decrease in financial resources, mobility impairment and death of contemporaries, among others [18]. For this purpose, we have considered a panel data for a set of European countries and have used logistic regressions. Hereafter, using the panel structure of the data allows us to relax the homogeneity assumption and control for unobserved individual heterogeneity, as well as for potential differences between waves. Precisely, we have used data from five panel waves (Waves 1, 2, 4, 5 and 6) from the Survey on Health, Ageing and Retirement in Europe (SHARE). Moreover, as number of chronic diseases (among other) is not asked in Wave 3, this one is not considered in our study. In addition, we have extended the results for the full sample by considering three geographic macro-areas (Nordic, Continental and Southern). The hypotheses here postulated are: a) the three objective aspects considered as proxies of social isolation will be associated with chronic diseases after age 50; b) socio-demographic variables also matter where multiple chronic diseases are concerned; c) due to the divergences in both social networks and welfare regimes, the association between the variables included and multiple chronic diseases will be somewhat different across the geographic macro-areas considered in this analysis.

In doing so, we are making a distinction with previous contributions and we provide new highlights for chronic prevention in European countries. The data have been obtained from the SHARE, Waves 1 to 6 (2004–2015). Hence, this study explores the relationship of social isolation with chronic diseases. The findings contribute to the knowledge in the field of social isolation and health. The main manuscript strengths are: the sample size, the 5-waves follow-up, and the multi-country analyses. These strengths provide enriched information in order to better understand the different relationships.

This paper is organized as follows. In the subsequent section, we describe the data sources we have used along with the methodological decisions we have taken based on the SHARE longitudinal survey. In addition, we present the empirical findings, while discussion and main conclusions are contained in the final section.

## Material and methods

### Data sample

Data for the current analysis are based on the SHARE, which is a multi-national prospective cohort study of people, aged 50 and over. Based on probability samples, SHARE is designed to be representative of the older community-dwelling population across different European countries (it covers 27 European countries plus Israel). Participants have been interviewed biennially since 2004.

Nonetheless, our eligible sample is restricted by data availability. Panel data from Waves 1–6 (2004–2015) are used in this study and countries included in the full sample represent Nordic (Denmark and Sweden), Continental (Austria, Belgium, France, Germany and Switzerland) and Southern (Italy and Spain) European countries. Therefore, these individuals from our analytical sample are not from all of the sample countries that comprise the SHARE project. Traditionally, studies have classified countries by clusters corresponding to geographic macro-areas. Besides, these areas correspond to Welfare Regimes: Social-democratic, Continental and Mediterranean [19–20]. In this analysis, we follow this classification. Table 1 contains the sample distribution by country and geographic macro-area, respectively.

**Table 1:** Distribution of the analytical sample by country and geographic macro-areas (all countries (9); sample size (n) = 37,864).

Country/Area	Wave 1	Wave 2	Wave 3	Wave 4	Wave 5	Wave 6	*Total* *Waves*
*Austria*	357	365	367	368	368	368	*2*,*193*
*Belgium*	1,308	1,330	1,341	1,348	1,351	1,354	*8*,*032*
*Denmark*	575	605	611	615	616	618	*3*,*640*
*France*	671	683	690	693	695	696	*4*,*128*
*Germany*	537	545	545	547	548	549	*3*,*271*
*Italy*	911	926	930	932	932	933	*5*,*564*
*Spain*	674	684	687	690	692	692	*4*,*119*
*Sweden*	753	763	766	766	766	766	*4*,*580*
*Switzerland*	377	389	391	393	393	394	*2*,*337*
*Nordic*	1,328	1,368	1,377	1,381	1,382	1,384	*8*,*220*
*Continental*	3,250	3,312	3,334	3,349	3,355	3,361	*19*,*961*
*Southern*	1,585	1,610	1,617	1,622	1,624	1,625	*9*,*683*
***Total***	*6*,*163*	*6*,*290*	*6*,*328*	*6*,*352*	*6*,*361*	*6*,*370*	*37*,*864*

*Source*: Authors’ calculations based on easySHARE release 6.0.0 (Waves 1 to 6: 2004–2015). Population aged ≥ 50. Macro-areas: (i) Nordic (Denmark and Sweden), (ii) Continental (Austria, Belgium, France, Germany and Switzer*land)*, *(iii) Southern (Italy and Spain)*.

Notes: Number of chronic diseases is not asked in Wave 3.

Therefore, our sample is restricted to population aged ≥ 50 (n = 282,297). We have taken age 50+ as the starting point of the SHARE. Besides, four groups: 50–59 years, 60–69 years, 70–79 years and ≥ 80 years have been contemplated in estimates to determine whether there are differences. Besides, as we have excluded individuals who did not respond in consecutive SHARE Waves (those lost over follow-up data n = 244,430), our analytical sample is composed of 37,864 individuals. Moreover, as the number of chronic diseases is not asked in Wave 3, the final sample in estimates is based on 31,536 observations distributed as follows by macro-areas: 6,843; 16,627 and 8,066 observations for Nordic, Continental and Southern, respectively.

### Measurements

**Fig 1:**
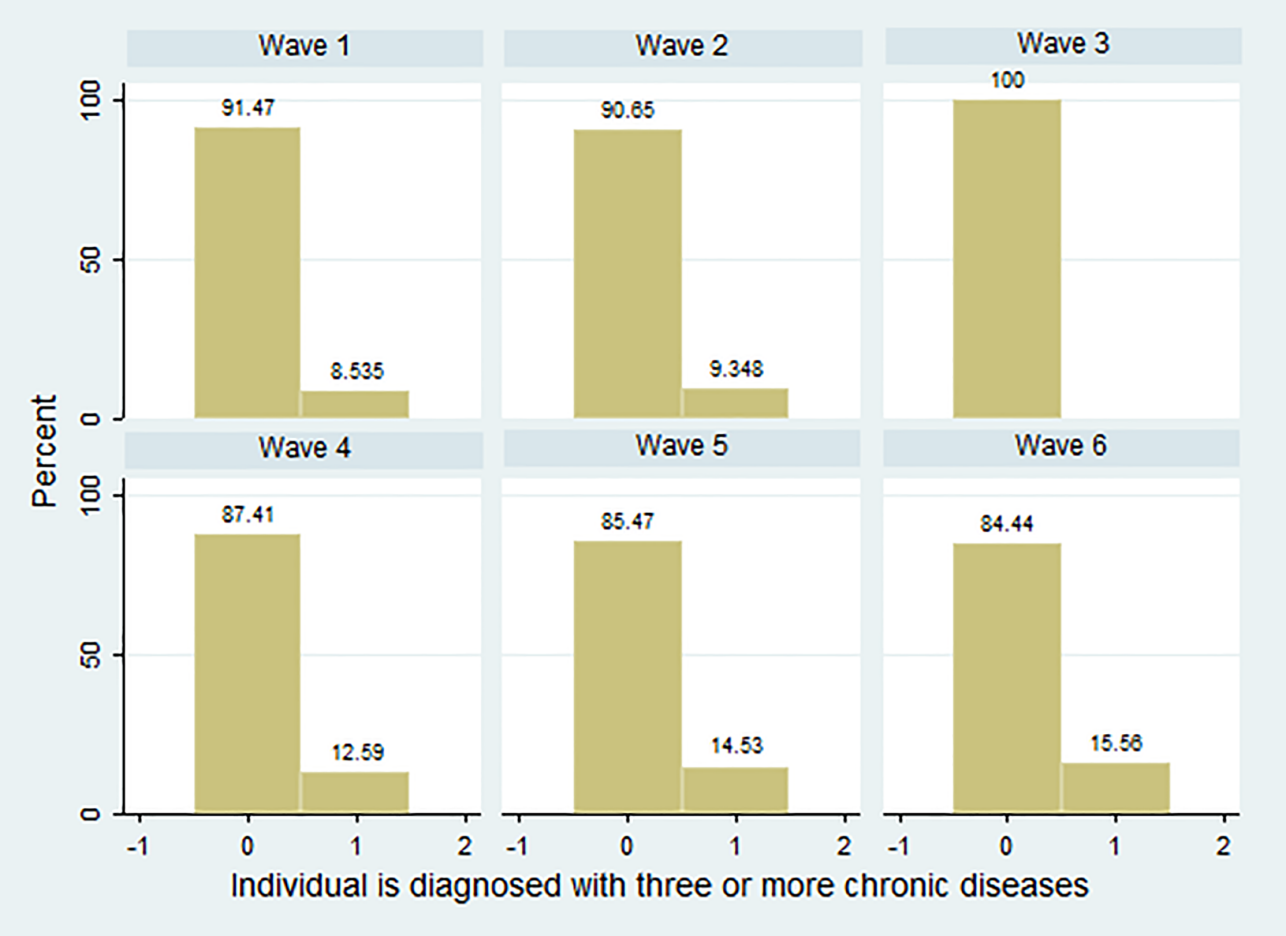
Distribution (percentages) of *Chronic* by SHARE Wave. *Source*: Authors’ calculations based on easySHARE release 6.0.0 (Waves 1 to 6: 2004–2015). Population aged ≥ 50. Macro-areas: (i) Nordic (Denmark and Sweden), (ii) Continental (Austria, Belgium, France, Germany and Switzerland), (iii) Southern (Italy and Spain). *Notes*: Number of chronic diseases is not asked in Wave 3.

All variables used in estimates are at an individual level and cover the entire relevant aspects (see Table 2). On the one hand, as a dependent variable, we consider *Chronic* to be a binary one. It takes value 1 if the person is diagnosed with three or more chronic diseases and zero otherwise. That is, while it may not be surprising that more Europeans have a chronic condition, what is striking (see Fig 1) is the increasing number of people that have multiple chronic conditions (MCCs). Table 2 presents these percentages, for the full sample and distinguishes according to geographic macro-area. It will be observed that although the performance is similar between the full sample and each macro-area, higher percentages are presented in Southern European countries, Italy and Spain, for the whole period considered.

**Table 2:** Distribution (percentages) of *Chronic* in the analytical sample by SHARE Wave and geographic macro-area (all countries (9); sample size (n) = 37,864).

Wave	Full sample	Nordic	Continental	Southern
*1*	8.53	8.21	7.85	10.22
*2*	9.35	10.16	8.54	10.31
*4*	12.59	12.17	12.00	14.18
*5*	14.53	13.24	13.06	18.66
*6*	15.56	15.53	14.07	18.65

*Source*: Authors’ calculations based on easySHARE release 6.0.0 (Waves 1 to 6: 2004–2015). Population aged ≥ 50. Macro-areas: (i) Nordic (Denmark and Sweden), (ii) Continental (Austria, Belgium, France, Germany and Switzerland), (iii) Southern (Italy and Spain).

*Notes*: Number of chronic diseases is not asked in Wave 3.

As control variables, we explore both *social isolation measurements* and *socio-demographic variables* (using for all dummy variables). *Social isolation measurements*: through three proxies. *Alone* takes value 1 if respondent lives alone. As marital status and living arrangements are clearly correlated, we do not use any socio-demographic variables related with marital status or civil partnership; *Help*, which takes value 1 if the activities of the individual during the last month include providing help to family, friends or neighbours; and *Club* codified as 1 when the activities for individuals in the previous month include going to sports, social or other clubs. *Socio-demographic variables* are control variables related to well-being, 1 if low Quality of Life (QoL). QoL is a common measurement used for well-being, CASP-12 in SHARE data. It is based on four subscales of control, autonomy, pleasure and self-realization. It ranges between 12 and 48 and it is interpreted as follows: low QoL, <35; moderate, 35–37; high, 37–39; and very high, ≥ 39. Because multicollinearity problems could appear in estimates, other health variables, in spite of being available in the survey (such as self-assessed health) are excluded in our final model. Furthermore, gender (1 if female); age (four levels: 50–59, 60–69, 70–79 and ≥ 80); educational level (measured according to international classification ISCED-97: low, middle and high education); employment status (unemployed, employed, retired and disabled—given the nature of the sample, individuals aged ≥ 50, there are not enough observations to sort by type of job) and geographic characteristics (*Rural* is 1 whether the person lives in a rural area or not) are considered.

### Analysis

Our dependent variable *y* is a binary one, and so, takes a value of 1 if the individual has a characteristic, here if the person is diagnosed with three or more chronic diseases (with probability *p*) and 0 otherwise (with probability (1 − *p*)). The expected value of *y*, *Ey* = 1 ⋅ *p* + 0 ⋅ (1 − *p*) = *p*, is the probability that MCCs occur. We assume that this probability is a function of a vector of explanatory variables (*x*) and a vector of unknown parameter *β*. Then, we use discrete choice models as follows:

Prob(y=1)=F(x,β), (1)

Prob(y=0)=1−F(x,β) (2)

The most common specifications for discrete choice models are probit and logit models. So, a latent variable interpretation from Eqs (1) and (2) leads to:

$$y=1\;if\;y_{i}^{*}>0$$ (3)

$$y=0\;if\;y_{i}^{*}\leq 0,$$ (4)

$$\mathrm{where}\;y^{*}=x^{\prime }\beta +\varepsilon ,$$ (5)

and *ε* is the error term.

Therefore, logit/logistic regression models are used to study the impact of social isolation proxies and socio-demographic variables on MCCs among the oldest people for a sample of European countries included in the SHARE.

In the logit model, the conditional probability is described by the cumulative logistic distribution (conditional to some explanatory variables *X*) the predicted probabilities always being between zero and one:

$$p=Prob\;(y=1|X)=\frac{\exp\left(X^{\prime }\beta \right)}{1+\exp\left(X^{\prime }\beta \right)}$$ (6)

The logit (log of odds) can be expressed as follows:

$$\ln\left(\frac{p}{1-p}\right)=X’\beta$$ (7)

The logit model is nonlinear and the sign of the estimates determines the direction of the relationship between variables. However, to interpret coefficients it is useful to introduce the odds ratios, understood as the ratio of the probability of success and the probability of failure. That is, an exponential function of fitted F(X’β). These empirical results are presented in the following section.

## Results

In this section, we present the main findings from our empirical research based on our three social isolation proxies and socio-demographic variables. To fully understand these effects, in Table 3 we first use descriptive statistics to get some idea of what our data look like. Moreover, Table 4 reports the main estimates from our model.

**Table 3:** Descriptive statistics of the analytical sample by geographic macro-area (all countries (9); sample size (n) = 37,864).

Variable	Full sample		Nordic		Continental		Southern	
	Total (%)	Chronic (%)	Total (%)	Chronic (%)	Total (%)	Chronic (%)	Total (%)	Chronic (%)
*Alone*	22.94	30.03	27.62	37.96	26.06	32.69	12.56	20.27
*Help*	8.68	6.14	19.97	9.09	9.24	7.46	3.05	1.98
*Club*	7.99	5.59	11.76	9.83	9.06	6.00	2.57	1.98
*Casp_low*	44.32	48.34	32.63	30.84	40.58	42.25	61.97	70.27
*Female*	57.33	58.84	56.09	55.28	57.25	58.35	58.55	62.11
*Age*^*a*^	67.89	72.36	67.68	72.90	67.65	72.05	68.57	72.47
*Loweduc*	55.77	56.20	42.82	38.08	47.19	44.62	84.46	87.29
*Mideduc*	24.91	26.90	28.93	39.43	30.10	32.20	10.81	9.71
*Higheduc*	18.73	16.43	27.69	21.99	21.92	22.42	4.54	3.01
*Unemployed*	1.79	1.23	1.14	0.98	2.02	1.40	1.86	1.12
*Employed*	16.64	5.51	26.42	9.09	16.00	4.86	9.67	4.04
*Retired*	50.56	71.66	51.56	83.05	52.75	76.07	45.19	56.70
*Disabled*	1.96	4.41	2.25	4.05	1.69	4.16	2.28	5.07
*Rural*	49.14	58.42	35.75	40.54	53.37	63.37	51.76	63.06

^a^Mean, in absolute terms not percentages.

*Source*: Authors’ calculations based on easySHARE release 6.0.0 (Waves 1 to 6: 2004–2015). Population aged ≥ 50. Macro-areas: (i) Nordic (Denmark and Sweden), (ii) Continental (Austria, Belgium, France, Germany and Switzerland), (iii) Southern (Italy and Spain).

*Notes*: Number of chronic diseases is not asked in Wave 3.

**Table 4:** Associations of social isolation, socio-demographic variables and chronic illnesses: logistic regressions models (odds ratios and 95% confidence intervals).

Independent variables		Full sample			Nordic			Continental			Southern		
		OR	95%CI		OR	95%CI		OR	95%CI		OR	95%CI	
*Alone*	Yes	1.20	[1.04–1.39]	**	1.37	[0.97–1.93]	*	1.00	[0.87–1.13]		1.46	[1.21–1.77]	***
	No	1.00			1.00			1.00			1.00		
*Help*	Yes	0.84	[0.68–1.04]	*	0.58	[0.34–0.97]	**	0.93	[0.76–1.14]		0.79	[0.50–1.24]	
	No	1.00			1.00			1.00			1.00		
*Club*	Yes	0.70	[0.54–0.92]	***	0.82	[0.49–1.37]		0.65	[0.52–0.82]	***	0.89	[0.56–1.40]	
	No	1.00			1.00			1.00			1.00		
*Casp_low*	Yes	2.42	[1.54–3.79]	***	3.36	[2.24–5.02]	***	2.00	[1.75–2.29]	***	2.02	[1.71–2.40]	***
	No	1.00			1.00			1.00			1.00		
*Female*	Yes	0.96	[0.86–1.07]		0.75	[0.55–1.03]	*	1.00	[0.89–1.12]		1.01	[0.86–1.18]	
	No	1.00			1.00			1.00			1.00		
*Age*	50–59 years	1.00			1.00			1.00			1.00		
	60–69 years	2.19	[1.46–3.29]	***	2.70	[1.37–5.30]	***	1.97	[1.55–2.50]	***	1.74	[1.33–2.30]	***
	70–79 years	4.74	[2.18–10.32]	***	7.39	[3.31–16.51]	***	3.30	[2.52–4.33]	***	3.42	[2.50–4.69]	***
	≥ 80 years	5.70	[2.36–13.74]	***	10.81	[4.53–25.82]	***	4.06	[3.00–5.49]	***	3.29	[2.34–4.62]	***
*Education*	Loweduc	1.00			1.00			1.00			1.00		
	Mideduc	0.99	[0.88–1.11]		1.35	[0.94–1.93]	*	0.89	[0.78–1.01]	*	1.05	[0.84–1.31]	
	Higheduc	0.81	[0.68–0.97]	**	0.54	[0.36–0.82]	***	0.89	[0.77–1.03]		0.75	[0.52–1.10]	
*Employment status*	Unemployed	1.00			1.00			1.00			1.00		
	Employed	0.38	[0.24–0.60]	***	0.19	[0.07–0.51]	***	0.45	[0.34–0.59]	***	0.57	[0.40–0.82]	***
	Retired	1.06	[0.95–1.23]		1.07	[0.47–2.47]		1.19	[1.01–1.40]	**	0.88	[0.74–1.04]	
	Disabled	3.71	[1.78–7.76]	***	3.99	[1.26–12.63]	**	3.85	[2.65–5.59]	***	1.83	[1.27–2.62]	***
*Rural*	Yes	0.92	[0.93–1.03]		0.78	[0.58–1.08]		0.97	[0.86–1.09]		1.02	[0.89–1.18]	
	No	1.00			1.00			1.00			1.00		
*McKelvey & Zavoina’s R*^*2*^		0.15			0.18			0.16			0.13		
*Observations*		31,536			6,843			16,627			8,066		

*Source*: Authors’ calculations based on easySHARE release 6.0.0 (Waves 1–6: 2004–15). Population aged ≥ 50. Macro-areas: (i) Nordic (Denmark and Sweden), (ii) Continental (Austria, Belgium, France, Germany and Switzerland), (iii) Southern (Italy and Spain).*Notes*: N° chronic diseases is not asked in Wave 3. Sample: 31,536 observations (we dropped Wave 3).

***,** and * indicate significance at 1%, 5%, 10%

In this regard, descriptive statistics for the analytical sample are shown in Table 3. Precisely, the sample with full data is based on 37,864 individuals, 57.33% females with an average age of 67.89. Hence, Table 3 is the first approximation to determine both, the main risk factors associated with MCCs and potential divergences by areas (e.g. Southern participants are the eldest and have higher percentages of females). Prevalence among responders shows differences when looking for *Chronic*. Overall, as expected for all the samples considered, social isolation increases the risk of being diagnosed with multiple chronic illnesses. The same applies for lower quality of life, the higher the age, the lower the education level or being inactive, and to a certain extent for females.

Empirical findings for the logistic panel models are presented in Table 4 using odds ratios. Thus, Column 1 includes the variables and Column 2 describes the detailed findings for the full sample. The following ones do so for each of the geographic macro-areas. It can be seen that coefficients are statistically significant and have the expected signs according to a priori criteria and as advanced in Table 3.

Concerning the first OR results column, statistically significant effects are obtained for all social isolation proxies. Consequently, the 1.20 odds ratio means that MCCs odds after age 50 are 20% higher for people living alone. However, people who provide relatives, friends and/or neighbours with help have a lesser probability of suffering from MCCs (OR = 0.84, 95% CI 0.68, 1.04). But as this proxy is statistically significant at 10%, it should be used and interpreted with caution. The same applies for those that participate in activities related to clubs (OR = 0.70, 95% CI 0.54, 0.92). As for socio-demographic variables, suffering MCCs increases with low well-being, age and being disabled (95% C.I.: 1.46 to 13.74). Besides, the reverse effect is shown for those with high education and being employed (95% C.I.: 0.24 to 0.97). Nonetheless, the gender and rural variables are not significant.

Turning to macro-areas OR results columns, our findings to a certain extent present changes with the aforementioned ones, mainly related with the significance of variables and not with constant direction. Formerly, whereas living alone (OR = 1.37, 95% CI 0.97, 1.93) and help (OR = 0.58, 95% CI 0.34, 0.97) isolation proxies are significant for the Nordic macro-area, the latter is only significant at 10%, and so, must be interpreted with care. Club-participation and living alone ones are significant for Continental and Southern macro-areas, respectively (OR = 0.65, 95% CI 0.55, 0.82; OR = 1.46, 95% CI 1.21, 1.77). Besides, it is worth noting that the educational factor is not relevant for Southern ones. Moreover, results are just about unchanging between geographic macro-areas when considering low well-being, age and employment status. Again, factors associated with rurality are not statistically significant.

## Discussion

NCDs undermine social and economic development, and so, constitute a basic public health challenge these days in developed countries [21]. Following the linkages outlined by previous studies and although specific mechanisms connecting socioeconomic determinants and NCDs have not been examined quantitatively, in this study we focus on the elderly population (aged ≥ 50; it is known that in the later years of life unwanted loneliness and social isolation occur more). Precisely, attention is put on “the newest” risk factors for NCDs, that is, those linked to social isolation.

From the presentation above, and in spite of the fact that different socio-demographic variables are significant regarding the propensity of being diagnosed with chronic illnesses, it should be clear that elderly Europeans with lesser social isolation have lower risk of suffering from MCCs. Social isolation and loneliness are intrinsically related but to a certain degree distinct concepts that should in all cases be considered [22]. Indeed, our empirical findings confirmed these issues related with social isolation and should be contemplated in the development of new public policies.

Therefore, the study raises societal questions as to why social isolation might be related with health status, and so, points to a way to reduce the increase in healthcare expenditures. Intuitively, it is to be expected that if a person with greater social participation has a lower risk of suffering from multiple chronic diseases, this would be associated with low health care utilization [23–24]. As previously claimed by Becchetti et al. [25], the identification of the different factors, as well as their heterogeneous impact across different population groups is decisive when tackling the challenge of improving health outcomes without threatening the sustainability of health care systems. Therefore, along with the traditional risk factors associated with lifestyles [26], “the newest” risks linked to isolation should be taken into consideration [27–28].

Moreover, as social isolation among older Europeans changes by country [29–30], we have studied three geographic macro-areas (Nordic, Continental and Southern). Hence, using logistic panel models, we have tested differences between the importance of living alone, club activities and providing family or neighbours with help in preventing or alleviating MCCs by macro-areas. In this regard, it is known than in Southern Europe, the majority of caring responsibilities for the elderly fall to the family whereas in Nordic countries they are largely supported by the State [31]. In this manuscript, it has been observed that whereas living alone is significant for Southern macro-areas, providing help is significant for the Nordic macro-area, with the club proxy being the main one for Continental ones. Thus, different interventions and tools should be considered in each area.

## Conclusion

Grounded in empirical research, our findings from this study contribute to a better understanding of social isolation in the following ways. Firstly, the longitudinal logit model estimated affords insights into the extent that social isolation is implicated with increasing multiple chronic diseases. Secondly, this risk linked to social isolation should be considered in different ways according to each area (or country). As was previously indicated in Rico-Uribe et al. [31], both socio-economic factors as well as several characteristics of welfare systems could be behind the observed differences across areas or countries. For this reason, the development of new public policies is required in each area depending on their corresponding characteristics. In this regard, addressing the transverse effects associated with aging could be a key point. A result that was previously found in several studies [32–33] shows that there is a significant positive effect between improvements in social relationships and individual health status. What is more, these findings are similar to the results in the literature regarding social isolation, providing help, living alone, and participation in club activities [15, 34–35]. Accordingly, our findings suggest that there are generic approaches aimed at improving social relationships (and hence, individual well-being) and that it is important to develop a more tailored approach in order to achieve better health outcomes.

However, a few limitations and extensions should also be mentioned. Firstly, in spite of working with micro data we should not forget that this is self-reported information, and so, recommendations from our findings and the corresponding policy implications should be taken with all due caution. Moreover, further studies are required to explore specific illnesses and more social isolation proxies. Although we have considered three objective aspects as proxies of social isolation, we have to recognize that they are also relative measurements with dichotomized scales. In addition, even though associations have been adjusted for socioeconomic measurements, other possible mechanisms or confounders could be behind our estimates. Reverse causality, and chronic diseases leading to social isolation, could also explain some of our findings [36]. Besides, when more data is available, it would be interesting to study more differences between and within countries in order to gain a better understanding for public policies.

Despite the above-mentioned limitations, we can postulate that this study provides new and valid information on the impact of social isolation and multiple chronic diseases after age 50. Therefore, together with the traditional risks factors associated with lifestyles, we suggest that it may be useful for researchers and policy makers to focus on social displacement. We really think that the implementation of well-informed public measures by policy makers to guard their populations against social isolation environments is useful. Hence, the preceding literature has identified two main types of interventions: group-based interventions and one-to-one interventions [37]. But they can be established both in community centres and at patients’ homes, and should focus on social skins (e.g. educational courses on social behaviours), social support (e.g. volunteer programs) and/or social interaction (e.g. providing services like transportation or internet use). Taking advantage of the opportunities that come with these factors, will determine Welfare States’ success.
